# Effects of deep neuromuscular block with low-pressure pneumoperitoneum on respiratory mechanics and biotrauma in a steep Trendelenburg position

**DOI:** 10.1038/s41598-021-81582-0

**Published:** 2021-01-21

**Authors:** Ji Eun Kim, Sang Kee Min, Eunji Ha, Dongchul Lee, Jong Yeop Kim, Hyun Jeong Kwak

**Affiliations:** 1 Department of Anesthesiology and Pain Medicine, Ajou University School of Medicine, 164, World cup-ro, Yeongtong-gu, Suwon, Republic of Korea; 2 Department of Anesthesiology and Pain Medicine, Gil Medical Center, Gachon University College of Medicine, 774, Namdong-daero, Namdong-gu, Incheon, Republic of Korea

**Keywords:** Endocrinology, Medical research, Signs and symptoms

## Abstract

We hypothesized that deep neuromuscular blockade (NMB) with low-pressure pneumoperitoneum (PP) would improve respiratory mechanics and reduce biotrauma compared to moderate NMB with high-pressure PP in a steep Trendelenburg position. Seventy-four women undergoing robotic gynecologic surgery were randomly assigned to two equal groups. Moderate NMB group was maintained with a train of four count of 1–2 and PP at 12 mmHg. Deep NMB group was maintained with a post-tetanic count of 1–2 and PP at 8 mmHg. Inflammatory cytokines were measured at baseline, at the end of PP, and 24 h after surgery. Interleukin-6 increased significantly from baseline at the end of PP and 24 h after the surgery in moderate NMB group but not in deep NMB group (*P*_group*time_ = 0.036). The peak inspiratory, driving, and mean airway pressures were significantly higher in moderate NMB group than in deep NMB group at 15 min and 60 min after PP (*P*_group*time_ = 0.002, 0.003, and 0.048, respectively). In conclusion, deep NMB with low-pressure PP significantly suppressed the increase in interleukin-6 developed after PP, by significantly improving the respiratory mechanics compared to moderate NMB with high-pressure PP during robotic surgery.

## Introduction

Laparoscopic surgery has become very popular due to less surgical stress response and better clinical effects including reduced operation time, bleeding, opioid requirement, and hospital stay, when compared to open surgery^1,2^. Recently, robotic surgery, which offers higher precision, faster recovery, and shorter hospitalization compared with laparoscopy, has gained popularity^3^. However, it requires prolonged pneumoperitoneum (PP) and steep Trendelenburg position, which can cause physiologic changes promoted by elevated intra-abdominal pressure (IAP), consequently giving rise to significant ischemia in abdominal organs and even in remote organs such as the lungs^4,5^. In addition, the cephalic elevation of the diaphragm markedly decreases the compliance of the respiratory system and tidal volume during the surgery.

International guidelines recommend the use of ‘the lowest IAP allowing adequate exposure of the operative field rather than a routine pressure^6^, and deep neuromuscular blockade (NMB), which improves surgical space conditions and facilitates the lowering of IAP, thus enhancing the satisfaction of the surgeon^7^. Because of the ability of sugammadex to antagonize rocuronium rapidly at any level of NMB, it is possible to continue the deep NMB until the end of surgery. In a meta-analysis, overall quality of evidence for advantages of low-pressure PP compared to high-pressure PP was evaluated^8^; however, these results did not consider the depth of NMB at all.

Ventilator-induced lung injury is caused by repetitive stress and/or strain on the lungs, which releases the mediators associated with activation of the immune response, further adding to lung injury and systemically causing distal organ dysfunction; this is termed biotrauma^9^. Pulmonary biotrauma is lessened by prone position, recruitment maneuver, and extracorporeal techniques^9,10^. Protective lung ventilation with low tidal volume and low positive end-expiratory pressure (PEEP) during laparoscopy reduced pulmonary complications^11^ but seemed to be not enough to reduce the biotrauma^12^. In a meta-analysis, early administration of an NMB agent was found to improve the outcomes by decreasing biotrauma in cases of acute respiratory distress syndrome (ARDS)^13^. Meanwhile, there is still no clinical research on deep NMB combined with low-pressure PP that would improve the respiratory condition and reduce inflammation during mechanical ventilation. We hypothesized that deep NMB combined with low-pressure PP would improve respiratory mechanics and then reduce biotrauma compared to moderate NMB combined with high-pressure PP during protective lung ventilation.

The aim of this study was to evaluate the effects of deep NMB combined with low-pressure PP and moderate NMB combined with high-pressure PP on respiratory mechanics and biotrauma during protective lung ventilation for robotic gynecologic surgery in a steep Trendelenburg position.

## Results

**Figure 1:**
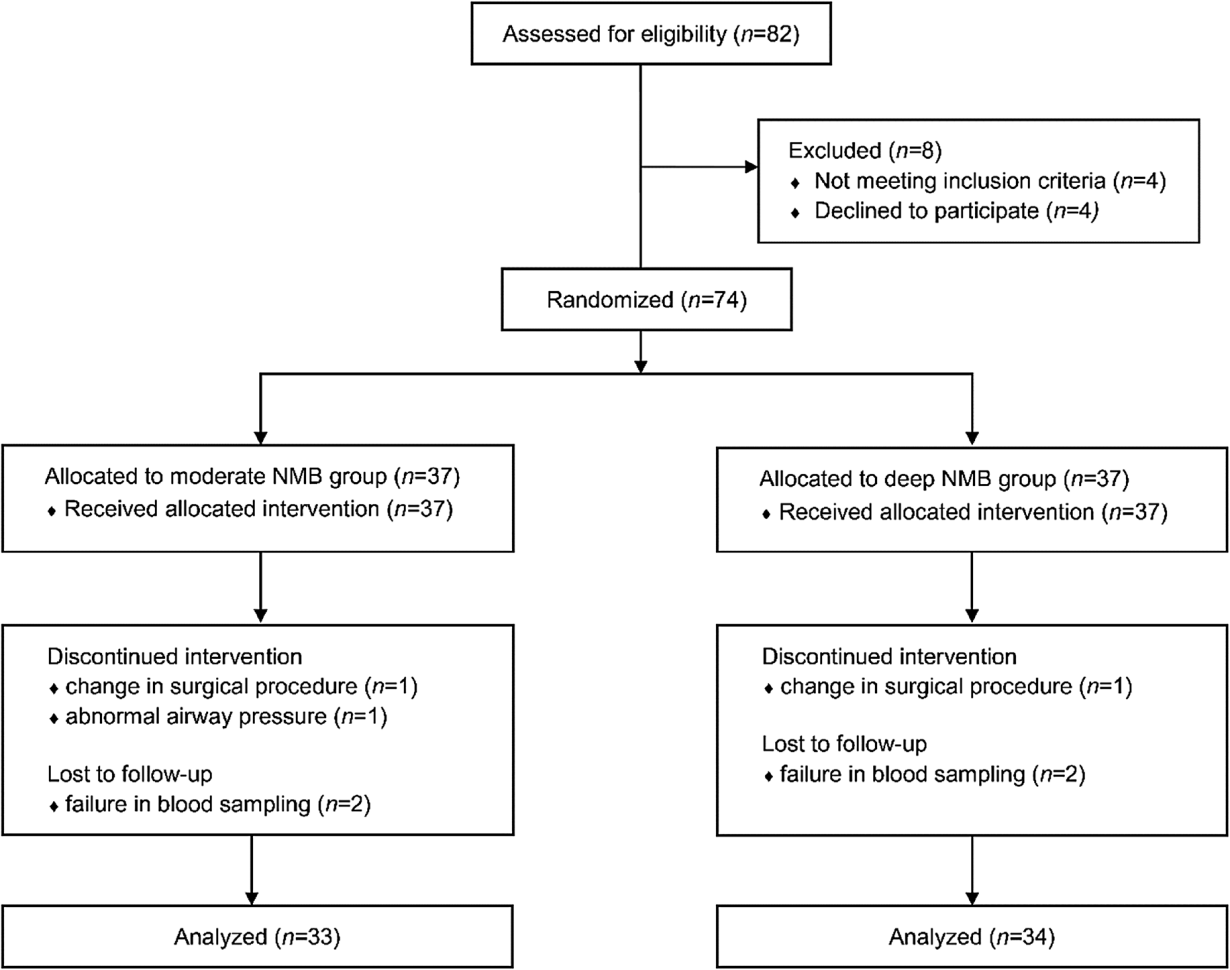
Patient allocation diagram. Abbreviations: *NMB* neuromuscular block.

A total of 74 patients were enrolled and randomized, but 7 patients were excluded because of a change in the surgical procedure (*n* = 2), abnormal airway pressure (*n* = 1), and failure in blood sampling (*n* = 4) (Fig. 1). There were no significant differences in patient characteristics and operation details (Table 1).

**Table 1:** Patients characteristics and operative details.

	Moderate NMB (*n* = 33)	Deep NMB (*n* = 34)
Age (year)	44 (9)	43 (7)
Weight (kg)	57 (9)	58 (11)
Height (cm)	158 (5)	158 (5)
Body mass index (kg/m^2^)	23 (4)	23 (4)
ASA physical status (1/2)	28/5	28/6
Type of operation	10/18/5	7/20/7
(Hysterectomy/myomectomy/others*)		
*Intraoperative*		
Propofol (mg)	1300 [700−2200 (1100−1500)]	1000 [400−3000 (1000−1300)]
Remifentanil (μg)	1906 [1000−4000 (1600− 2000)]	2000 [400−3000 (1400−2000)]
Urine output (mL)	100 [30−530 (65−160)]	90 [35−400 (60−125)]
Bleeding loss (mL)	100 [10−500 (30−100)]	100 [10−500 (50−130)]
Duration of operation (min)	143 (62)	129 (42)
Duration of anesthesia (min)	182 (69)	169 (49)
Duration of pneumoperitoneum (min)	100 [40−273 (73−120)]	90 [40−192 (75−120)]
Duration of hospital stay (days)	4 [2−7 (3−4)]	3 [2−5 (3−4)]

Values are presented as mean (standard deviation), median [range (interquartile range)], and the number of patients.

Abbreviations: *NMB* neuromuscular block, *ASA* American Society of Anesthesiologists, *RBC* red blood cell.

*Includes staging, salpingo-oophorectomy, cystectomy, and uterovaginal prolapse.

**Figure 2:**
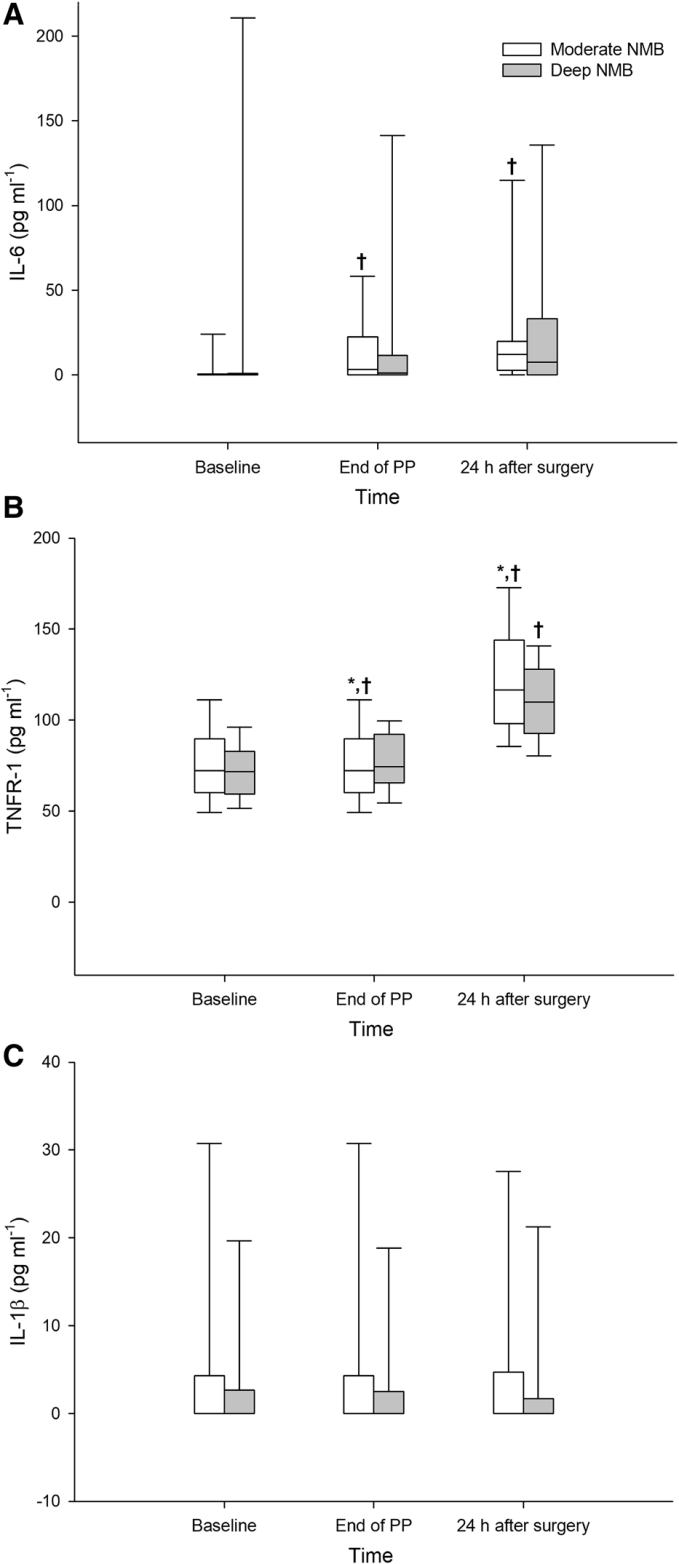
Changes in interleukin (IL)-6 (**A**), tumour necrosis factor receptor (TNFR)-1 (**B**), and IL-1β levels (**C**). Abbreviations: *NMB* neuromuscular block, *PP* pneumoperitoneum, Baseline, after anesthetic induction. The box plots represent the median, interquartile range, and 10th and 90th percentile (whiskers). *P < 0.05 between-group comparison. ^†^*P* < 0.05 versus baseline in each group.

In post hoc analysis of cytokines, IL-6 level increased significantly from baseline at the end of PP and 24 h after the surgery in the moderate NMB group (*P* = 0.014 and *P* = 0.001, respectively), whereas there was no significant change in the deep NMB group (Fig. 2). TNFR-1 level increased significantly from baseline after PP in both groups (*P* < 0.001 in all) and was significantly higher in the moderate NMB group than the deep NMB group at the end of PP and 24 h after the surgery (*P* = 0.038 and *P* = 0.046, respectively, Fig. 2). IL-10 was not detected at baseline in the deep NMB group, but the levels were similar between the two groups at the end of PP and 24 h after the surgery. IL-4 level at baseline was similar between the two groups, but IL-4 at the other 2 time points and TNF-α at all time points were not detected in both groups (all zero, data not shown).

There was a significant interaction between group and time in Ppeak, Pplat, Pdriving, and Pmean (Table 2). In post hoc analysis, Ppeak was significantly higher in the moderate NMB group than in the deep NMB group at 15 min, 60 min after PP, and at the end of surgery (*P* = 0.002, *P* < 0.001, and *P* = 0.035, respectively). In addition, Pplat and Pdriving were significantly higher in the moderate NMB group than in the deep NMB group at 15 min and 60 min after PP (*P* = 0.004 and *P* < 0.001, respectively). Moreover, Pmean was significantly higher in the moderate NMB group than in the deep NMB group at 15 min and 60 min after PP (both* P* = 0.005). IAP was adequately maintained at approximately 8 or 12 mmHg, according to the group, throughout PP without changing the pressure level.

**Table 2:** Intraoperative respiratory mechanics. Values are presented as mean (standard deviation).

	Baseline	15 min after PP	60 min after PP	End of surgery	*P*_group*time_ value
**Peak inspiratory pressure (cmH**_**2**_**O)**					0.002
Moderate NMB	16.7 (2.2)	26.9 (4.0)*^,†^	27.9 (3.7)*^,†^	19.7 (2.9)*^,†^	
Deep NMB	15.5 (2.7)	23.7 (4.0)^†^	23.9 (4.5)^†^	18.2 (3.0)^†^	
**Plateau airway pressure (cmH**_**2**_**O)**					0.003
Moderate NMB	14.2 (1.8)	23.8 (3.7)*^,†^	24.4 (3.1)*^,†^	16.6 (2.7)^†^	
Deep NMB	13.4 (1.9)	20.8 (4.4)^†^	21.0 (4.2)^†^	15.5 (2.8)^†^	
**Driving airway pressure (cmH**_**2**_**O)**					0.003
Moderate NMB	9.1 (1.8)	18.8 (3.7)*^,†^	19.4 (3.1)*^,†^	11.6 (2.7)^†^	
Deep NMB	8.4 (1.9)	15.8 (4.4)^†^	16.0 (4.2)^†^	10.5 (2.9)^†^	
**Mean airway pressure (cmH**_**2**_**O)**					0.048
Moderate NMB	8.5 (0.9)	11.0 (1.3)*^,†^	11.2 (1.3)*^,†^	9.1 (1.3)^†^	
Deep NMB	8.2 (0.8)	10.0 (1.4)^†^	10.2 (1.5)^†^	8.7 (1.5)^†^	
**Static lung compliance (mL/cmH**_**2**_**O)**					0.623
Moderate NMB	41.0 (7.5)	20.3 (4.0)*^,†^	19.5 (3.5)*^,†^	33.4 (7.5)^†^	
Deep NMB	43.8 (7.5)	25.2 (9.1)^†^	23.9 (5.4)^†^	36.0 (7.2)^†^	
**Dynamic lung compliance (mL/cmH**_**2**_**O)**					0.442
Moderate NMB	32.2 (7.7)	17.4 (3.6)*^,†^	16.5 (3.3)*^,†^	26.3 (6.4)^†^	
Deep NMB	36.2 (10.0)	20.4 (4.1)^†^	20.0 (4.2)^†^	28.7 (6.6)^†^	

Baseline means after anesthetic induction. Driving airway pressure means the pressure gradient from plateau pressure to positive end-expiratory pressure. Abbreviations: *NMB* neuromuscular block.

*P < 0.05 between-group comparison. ^†^*P* < 0.01 versus ‘baseline’ in each group.

White blood cell counts and CRP levels were similar between the two groups (Table 3). The number of patients with CRP > 0.5 mg/dL was similar in the moderate and deep NMB groups [26 (79%) vs 30 (88%), *P* = 0.340]. The intergroup differences in changes in HR, MAP, pH, PaCO_2_, and PaO_2_/FiO_2_ ratio from baseline were comparable over time (*P*_group*time_ = 0.566, 0.845, 0.904, 0.629, and 0.189, respectively) (data not shown).

**Table 3:** Postoperative data. Values are presented as mean (standard deviation), median [range (interquartile range)], and the number of patients. Abbreviations: NMB, neuromuscular block.

	Moderate NMB (*n* = 33)	Deep NMB (*n* = 34)	*P* value
**White blood cell count (μ/L)**			
Preoperative	6219 (1468)	5585 (1575)	0.115
24 h after surgery	8838 (2585)	8044 (1832)	0.215
C-reactive protein (mg/dL)	1.1 [0.2−5.6 (0.6−2.2)]	0.0 [0.2−3.6 (0.7−1.7)]	0.649
Chest X-ray	17/2/3	17/2/3	> 0.999
*(Pneumoperitoneum/pleural effusion/atelectasis)*			

## Discussion

This randomized controlled trial is the first study to evaluate the effect of deep NMB combined with low-pressure PP on respiratory mechanics and biotrauma during protective lung ventilation against PP. We demonstrated that in patients undergoing robotic gynecologic surgery, deep NMB combined with low-pressure PP significantly suppressed the increase in IL-6 level developed after PP, by significantly improving the respiratory mechanics compared with moderate NMB combined with high-pressure PP.

Increased IAP itself causes inflammatory-induced lung damage after PP^8,14^, although laparoscopy has clear benefits in terms of reduced inflammatory response compared to open surgery^6,15^. Recently, it has been reported that IL 1-β, IL-6, and TNF-α levels increased in lung tissue as IAP increased, which was confirmed by histologic examination^14^. Among randomized controlled trials in humans, Schietroma et al. observed a significant decrease in IL-1, IL-6, and CRP levels^16^, and Basgual et al. observed a lower increase in IL-6 level up to 24 h postoperatively in low-pressure PP compared to high-pressure PP^17^. However, Perrakis et al. and Torres et al. did not show any difference regarding levels of CRP, IL-6, IL-8, IL-10, and white blood cell count in low-pressure PP^18,19^. Rather, Vijayaraghavan et al. found a higher increase in CRP level at 24 h postoperatively in low-pressure PP^20^. However, these studies were performed in reverse Trendelenburg position and did not consider the depth of NMB.

In this study, we investigated the changes in inflammatory markers in moderate and deep NMB states by adding the concept of high- or low-pressure PP in female patients in the Trendelenburg position. As a result, IL-6 levels increased significantly at the end of PP and 24 h after the surgery from baseline in the moderate NMB group, whereas there was no significant change in the deep NMB group. Meanwhile, CRP, which is one of acute phase response proteins of inflammation^21^, was comparable at 24 h after surgery in our study. TNFR-1 mediates TNF-induced endothelial permeability, and its activation means compromised alveolar-capillary barrier and neutrophilic inflammation in the lungs^22^. In this study, TNFR-1 levels increased significantly from baseline after PP in both groups and was significantly higher in the moderate NMB group than in the deep NMB group at the end of PP and 24 h after the surgery, although there was no intergroup difference in the change in TNFR-1 level.

According to the detrimental impacts of PP on the blood circulation of intra-abdominal organs and cardiopulmonary functions^23^, low-pressure PP during laparoscopy has been tried with several benefits, but the evidence is weak yet^8^. In addition, low-pressure PP (< 12 mmHg) in gynecological laparoscopy worsened the visualization of the surgical field on account of needing higher pressures than usual laparoscopy clinically^24^. However, these studies were not conducted at the identical depth of the NMB^8,24^. Whereas, Martini et al. firstly reported that deep NMB improved the quality of surgical conditions than moderate NMB during laparoscopy without cardiorespiratory compromise, which was conducted at the identical retroperitoneal pressure^25^. In a meta-analysis, deep NMB was found to improve the surgical space conditions at both low- and high-pressure PP^7^.

PP increases ventilation-perfusion mismatch and decreases lung compliance and tidal volume through cephalic elevation of the diaphragm^6,26^, which are accentuated by the steep Trendelenburg position^27,28^. During gynecologic laparoscopy, the establishment of IAP 12 mmHg decreased the lung compliance up to 34% and increased Pplat and Ppeak by 6–7 cmH_2_O^26^, and change in IAP from 10 to 15 mmHg led to 11% decrease in lung compliance and 12% increase in both Pplat and Ppeak^28^. Ppeak is elevated by a combination of reduced dynamic and static compliance. As opposed to Ppeak, Pplat is considered as the most important determinant of barotrauma, because it may most accurately reflect the end-inspiratory volume and thus, lung injury. In addition, Pdriving is considered as the key determinant of lung injury. In a meta-analysis including 3562 ARDS patients, Pdriving was the strongest independent variable associated with survival among ventilation variables^29^. In our study, deep NMB combined with low-pressure PP significantly lowered Ppeak, Pplat, and Pdriving, and thus it might suppress the inflammatory responses associated with ventilator-induced lung injury as compared with moderate NMB combined high-pressure PP.

In a multicentre study (ACURASIS trial), early administration of an NMB agent, namely, cisatracurium, improved the outcome by decreasing biotrauma in ARDS^13^. It may be attributed to the anti-inflammatory properties of cisatracurium rather than to a consequence of the reduction in patient-ventilator asynchrony^30^. Although a new multicentre study (ROSE trial) detected no significant decrease on long-term mortality^31^, cisatracurium may reduce biotrauma and short-term mortality^32^. In our study, all patients were deep-sedated without asynchrony and received rocuronium as an aminosteroid NMB agent. In contrast to a report that rocuronium induced inflammation in vivo^33^, deep NMB plus low-pressure PP significantly reduced biotrauma in our study.

The current study has a few limitations. First, although the high IAP during mechanical ventilation increased inflammatory markers in the lungs^14^, the increase in systemic inflammatory cytokines might have resulted from other sites also. In rats, PP increased levels of IL-6 and TNF-α in the peritoneum or abdominal organs^34,35^. Second, all patients included in our study were female. Further validations are required in other gender populations. However, cytokines are related to the immune system; thus, changes in their concentrations induced by high pressure-PP or excessive CO_2_ absorption influence metabolism and phagocytotic ability of macrophages and decrease the cytotoxic activity of lymphocytes^15^. Therefore, our observation could be important, particularly in cancer patients, because immunosuppression is one of the factors responsible not only for postoperative infection but also for tumour spread and metastasis^36^.

In conclusion, the deep NMB combined with low-pressure PP significantly suppressed the increases in IL-6 level developed after PP, by significantly improving the respiratory mechanics compared to moderate NMB combined with high-pressure PP in a steep Trendelenburg position during robotic gynecologic surgery.

## Methods

After obtaining approval from the Ajou University Hospital Institutional Review Board (AJIRB-MED-OBS-18-115), this study was registered at http://clinicaltrials.gov (NCT 03576118). This study was performed in accordance with relevant guidelines and regulations. Between May 2018 and November 2019, 74 patients aged 25–80 years undergoing robotic gynecologic surgery with Trendelenburg position were enrolled. All participants provided written informed consent prior to randomization. The exclusion criteria were the presence of cerebrovascular disease, uncontrolled hypertension, asthma, neuromuscular disorder, a history of prior abdominal surgery, and morbid obesity (body mass index > 35 kg/m^2^).

### Interventions

Patients were randomly assigned to moderate or deep NMB groups using a computer-generated randomization technique (http://www.random.org). The moderate NMB group (*n* = 37) was maintained with a train of four (TOF) count of 1–2 and IAP at 12 mmHg during surgery and then reversed using glycopyrrolate (10 μg/kg) and neostigmine (50 μg/kg) after surgery. The deep NMB group (*n* = 37) was maintained with a post-tetanic count of 1–2 and IAP at 8 mmHg and then reversed using sugammadex (4 mg/kg). NMB was monitored using acceleromyography (TOF-Watch-SX; MSD BV, Netherlands), which was applied to the adductor pollicis muscle. The study intervention was conducted by an anesthetic provider who did not participate in the outcome assessment. Patients and outcome assessors of intraoperative and postoperative periods were blinded to the group assignment.

### Anesthesia

Standard monitoring, including pulse oximetry, electrocardiography, bispectral index (BIS), and noninvasive blood pressure measurement, was performed. Anesthesia was implemented as total intravenous anesthesia using target-controlled infusion of propofol and remifentanil. Anesthesia was induced with propofol of 4.0–6.0 μg/mL and remifentanil of 3.0–4.0 μg/mL as target concentrations. After loss of consciousness, the TOF-Watch-SX was calibrated and stabilized (< 5% variation in the TOF ratios). After administration of rocuronium (0.6 mg/kg) and subsequent confirmation of relaxation, tracheal intubation was performed. A 20-G radial arterial catheter was inserted for continuous monitoring of hemodynamics and blood sampling. Mechanical ventilation was composed of volume-controlled mode with a tidal volume 6 ml/kg of ideal body weight, an I:E ratio of 1:2, an external PEEP of 5 cmH_2_O, and an inspiratory pause of 10% (F_I_O_2_ = 0.5). Respiratory rate was set to an end-tidal carbon dioxide tension (EtCO_2_) between 30 and 40 mmHg. Ideal body weight was calculated according to a predefined formula for women: 45.5 + 0.919 × [height (cm) − 152.4]^37^. Anesthesia was maintained using propofol and remifentanil to achieve the BIS value of 40–60 and mean arterial pressure (MAP) within 20% of baseline. Rocuronium (0.3–0.4 mg/kg/h) was continuously infused and titrated according to the group assignment until the end of the fascia suturing. After dressing, the NMB was reversed, and extubation was done after confirming the TOF ratio > 0.9. Lactate Ringer’s solution or normal saline was infused at a rate of 6 ml/kg/h.

Trendelenburg position was set at 30°. PP was controlled by limiting CO_2_ insufflator. For patient-controlled analgesia, intravenous fentanyl was administered for 48 h at a rate of 0.4 µg/kg/h depending on the patient’s need.

### Data collection

Pro-inflammatory cytokine levels, including interleukin (IL)-6, tumour necrosis factor (TNF)-α, TNF receptor (TNFR)-1, and IL-1β, and anti-inflammatory cytokines, including IL-4 and IL-10, were measured. Blood samples were collected at 3 time points: after induction (baseline), at the end of PP, and 24 h after surgery; they were transferred to EDTA tubes and sent to the laboratory in a container. They were centrifuged at 3600 rpm for 30 min and 1.5 mL of the supernatant serum was collected in an Eppendorf tube which was subsequently frozen at − 80 °C for later analysis. The levels of cytokines were measured using a commercially available ELISA kit (R&D Systems, Minneapolis, Minnesota, USA). Each sample was analyzed in triplicates and excluded when at least one was not determined, and the average value was calculated.

Respiratory parameters, including peak inspiratory pressure (Ppeak), plateau airway pressure (Pplat), mean airway pressure (Pmean), and EtCO_2_, were recorded at 4 time points: after induction (baseline), 15 min and 60 min after PP, and at the end of surgery. Driving airway pressure (Pdriving) means pressure gradient from the plateau pressure to PEEP. The heart rate (HR), MAP, and parameters associated with arterial blood gas analysis (pH, PaO_2_, and PaCO_2_) were recorded at 5 time points: after induction (baseline), 15 min and 60 min after PP, at the end of PP, and at the end of surgery. The arterial blood samples were analyzed using a satellite blood-gas analyzer (Stat Profile pHOx Ultra, Nova Biomedical, Waltham, MA, USA). White blood cell count was measured preoperatively and 24 h after surgery. C-reactive protein (CRP, normal range ≤ 0.5 mg/dL) and chest X-ray were evaluated at 24 h after surgery.

### Statistical analysis

The primary endpoint was the change in the level of IL-6 during the surgery. The secondary endpoints were the changes in levels of other pro-inflammatory cytokines, anti-inflammatory cytokines, and respiratory parameters during the surgery. A previous study reported that the mean ± standard deviation (SD) value of IL-6 level was 45 ± 8.6 pg/L during mechanical ventilation after conventional PP^38^. Based on this previous result, we assumed that a 15% reduction in IL-6 level might be clinically significant. With an α-error of 0.05 and a β-error of 90%, 36 subjects were required in each group. We included 37 patients per group to allow for possible dropouts.

Values are expressed as mean (SD or standard error) or median [range (interquartile range)] or numbers of patients. The normality of distribution was assessed with the Kolmogorov–Smirnov test. Parametric data and nonparametric data were analyzed using the independent *t* test and the Mann–Whitney *U* test, respectively. Categorical variables were evaluated using the chi-square test or Fisher’s exact test. Intergroup comparisons for repeated-measures, including HR, MAP, pH, PaO_2_, PaCO_2_, respiratory parameters, and cytokines were performed using a linear mixed model with post hoc analyses as fixed effects. A *P* value < 0.05 was considered statistically significant. Statistical analysis was conducted using SAS (version 9.3, SAS Inc., Cary, NC, USA) and R package (version 3. 6.1).
